# Supplementary material for: Energy-Saving One-Step Pre-Treatment Using an Activated Sodium Percarbonate System and Its Bleaching Mechanism for Cotton Fabric
Source: Materials (Basel). 2022 Aug 25;15(17):5849. doi: 10.3390/ma15175849 (PMC9457162; doi:10.3390/ma15175849)
Supplement: Supplementary file 1 [file materials-15-05849-s001.zip › materials-1835462-supplementary.pdf]

Supplementary Material for

Energy-Saving One-Step Pre-Treatment Using an Activated Sodium Percarbonate System and Its Bleaching Mechanism for Cotton Fabric

Qing Li [1,2,3,†], Run Lu [1,†], Yan Liang [1], Kang Gao [1] and Huiyu Jiang [1,4,*]

[1] Hubei Key Laboratory of Biomass Fibers and Eco-Dyeing & Finishing, College of Chemistry and Chemical Engineering, Wuhan Textile University, Wuhan 430200, China
[2] Jiangsu Engineering Research Center of Textile Dyeing and Printing for Energy Conservation, Discharge Reduction and Cleaner Production, Soochow University, Suzhou 215123, China
[3] Key Laboratory of Clean Dyeing and Finishing Technology of Zhejiang Province, Shaoxing University, Shaoxing 312000, China
[4] National Innovation Center of Advanced Dyeing and Finishing Technology, Tai'an 271000, China
* Correspondence: dongminjiang@163.com
† These authors contributed equally to this work.

Contents of Supporting Information

4 figures in 5pages

Section Page

Figure S1	S2
Figure S2	S2-S3
Figure S3	S3-S4
Figure S4	S5

Figures

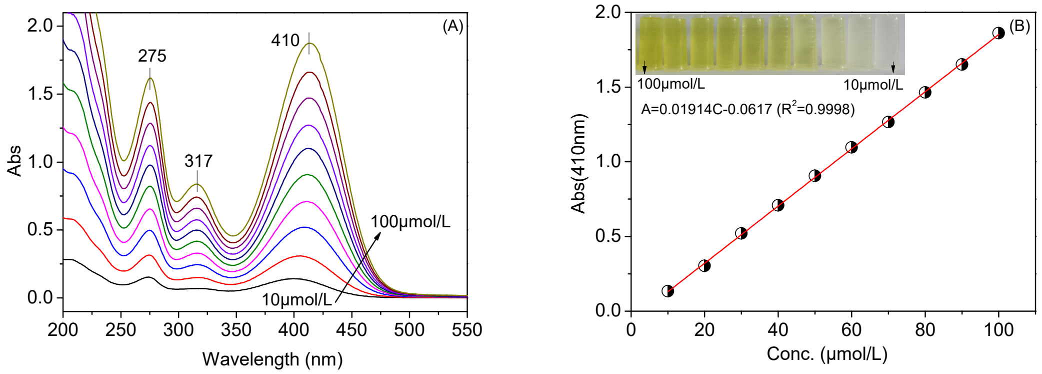

Figure S1 UV-Vis absorption spectrum (A) and standard curve (B) of morin solution

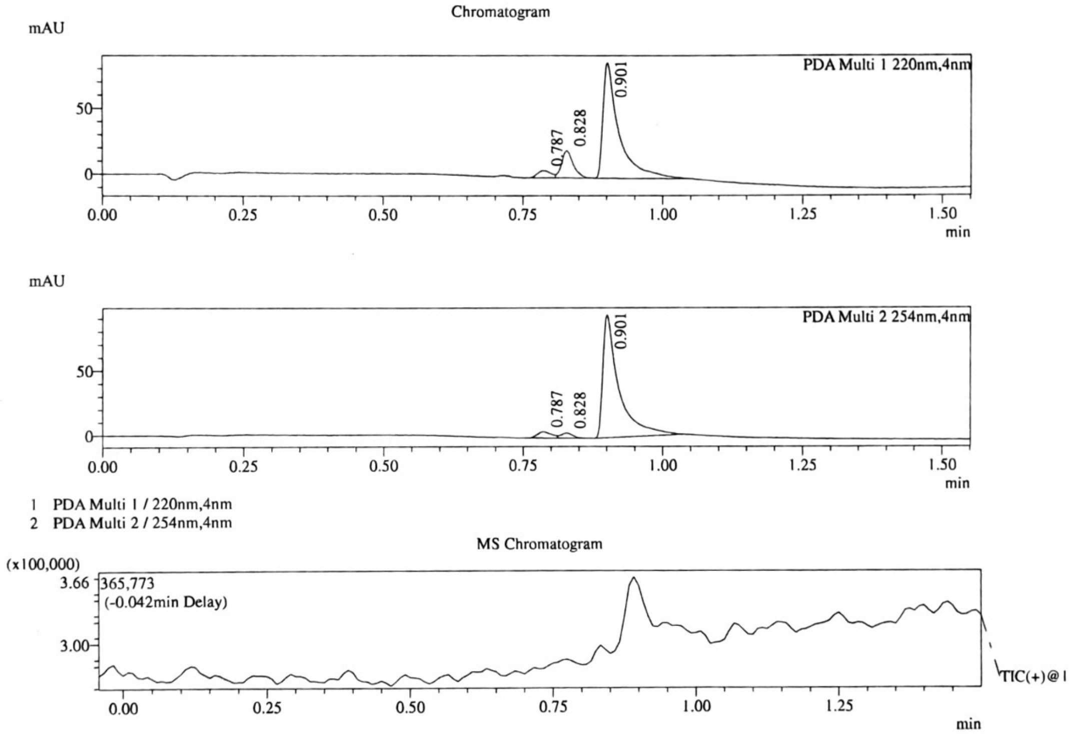

Integration Result

Peak Table

PDA Ch1 220nm

Peak#	Ret. Time	Height	Height%	USP Width	Area	Area%
1	0.787	5770	5.044	0.052	9765	4.530
2	0.828	20791	18.177	0.039	30067	13.946
3	0.901	87816	76.778	0.043	175757	81.524

Peak Table

PDA Ch2 254nm

Peak#	Ret. Time	Height	Height%	USP Width	Area	Area%
1	0.787	4814	4.639	0.051	8387	4.082
2	0.828	3912	3.769	0.042	5464	2.659
3	0.901	95047	91.592	0.043	191602	93.258

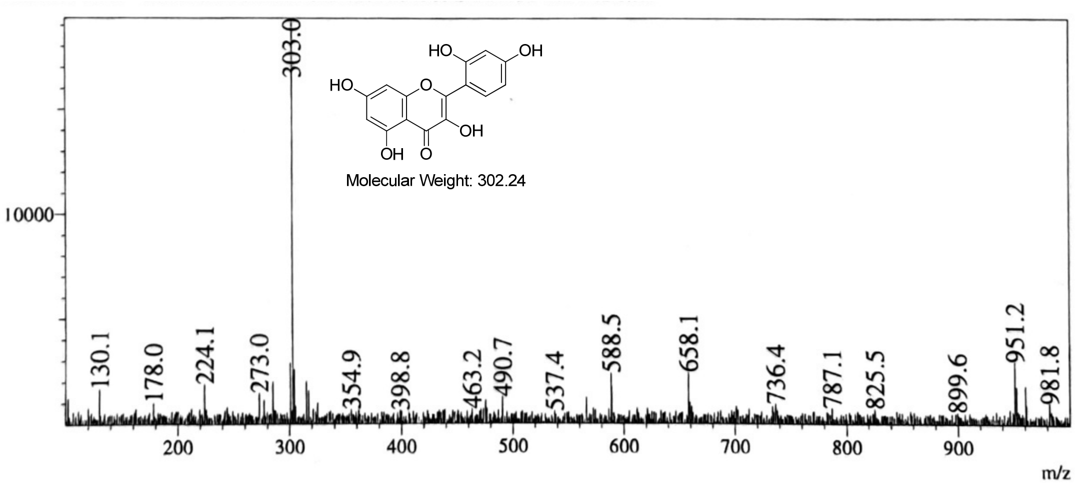

Figure S2 LC-MS of Morin (ESI+)

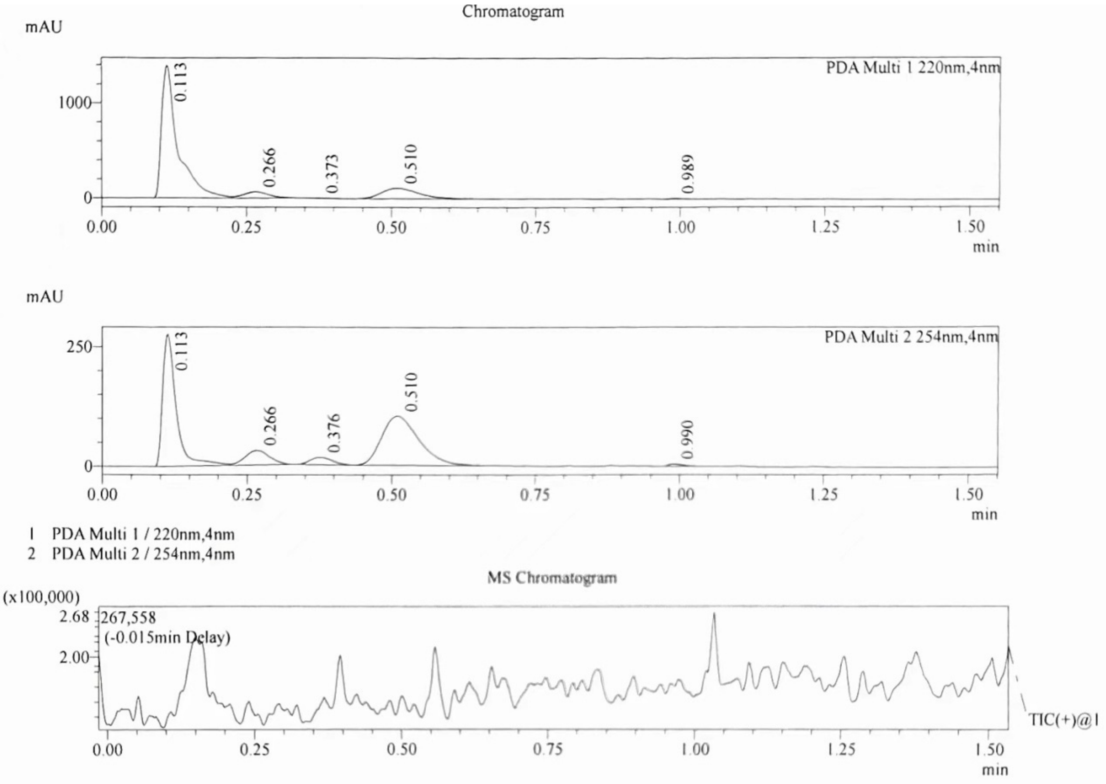

Integration Result

Peak Table

PDA Ch1 220nm

Peak#	Ret. Time	Height	Height%	USP Width	Area	Area%
1	0.113	1394238	88.335	0.041	2738235	79.196
2	0.266	63735	4.038	0.086	202109	5.845
3	0.373	3212	0.203	0.062	6663	0.193
4	0.510	110866	7.024	0.119	500742	14.483
5	0.989	6298	0.399	0.042	9803	0.284

PDA Ch2 254nm

Peak#	Ret. Time	Height	Height%	USP Width	Area	Area%
1	0.113	275887	63.956	0.041	451673	42.640
2	0.266	30980	7.182	0.080	92097	8.694
3	0.376	15647	3.627	0.075	42338	3.997
4	0.510	103418	23.974	0.119	465007	43.899
5	0.990	5435	1.260	0.040	8161	0.770

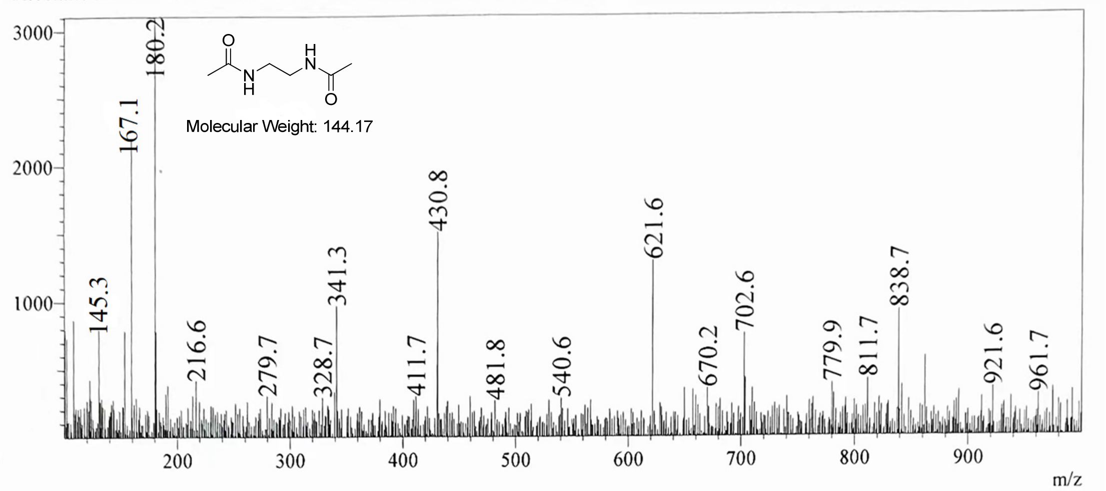

Figure S3 LC-MS of reaction solution (ESI+)

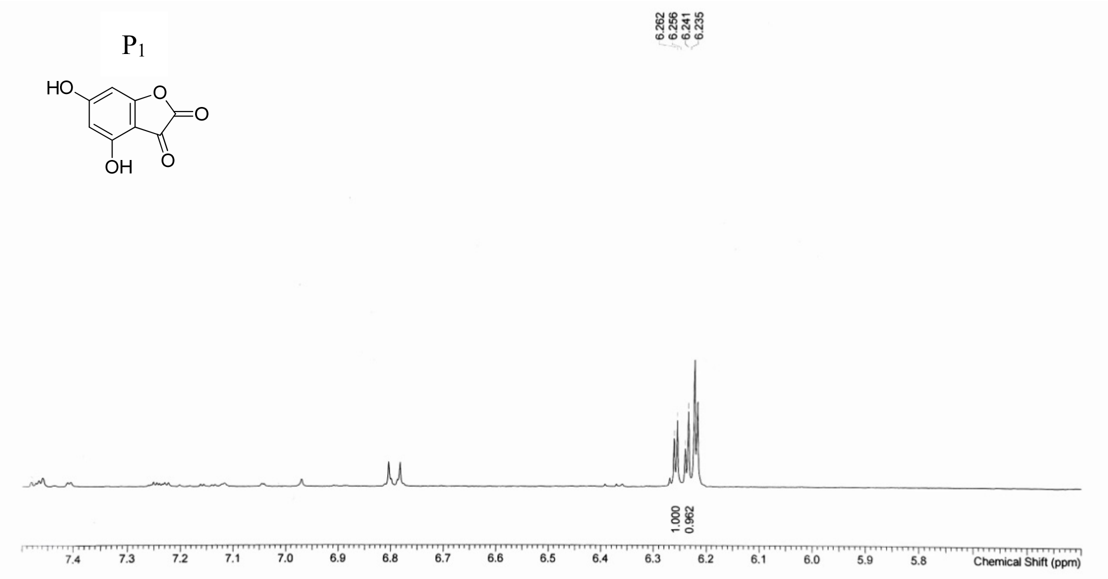

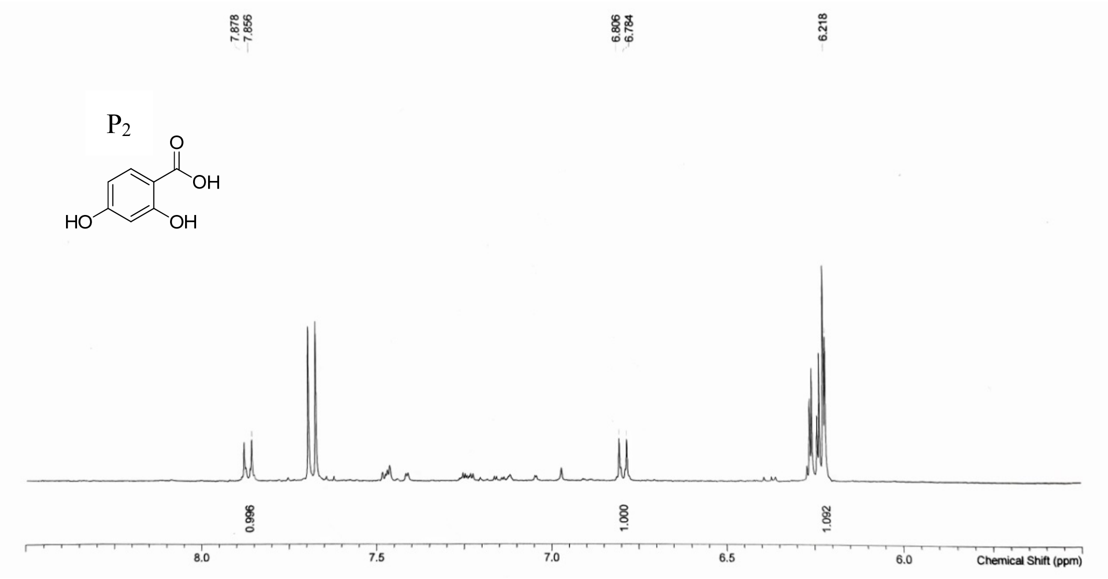

Figure S4[1] H-NMR of the degradation products of morin in SPC/TAED system
